# Supplementary material for: Hi-C calibration by chemically induced chromosomal interactions
Source: EMBO Rep. 2026 Apr 14;27(10):2549–58. doi: 10.1038/s44319-026-00772-x (PMC13219496; doi:10.1038/s44319-026-00772-x)
Supplement: Supplementary file 1 — Table EV1 [file 44319_2026_772_MOESM1_ESM.docx]

**Table EV1. Plasmid and Strain list**

| **Plasmid** | **Note** | **Note** |
| --- | --- | --- |
| pSR11 | 192X TetO with a TRP1 marker | Rohner *et al*.(Rohner *et al*, 2008) |
| pSR13 | 256X LacO with a LEU2 marker |  |
| pMY63 | REV1pr-LacI-GFP-REV1pr-TetR-mCherry with a HIS3 marker | Du *et al*.(Du *et al*, 2022) |
| pMY48 | REV1pr-LacI-FKBP12-REV1pr-TetR-FRB with an ADE2 marker |  |
| pSR13-S1 | pSR13 plasmid with homologous sequence from Chr15::433394-433698 | This study |
| pSR11-S3 | pSR11 plasmid with homologous sequence from Chr15::793783-794105 |  |
| pSR13-S4 | pSR13 plasmid with homologous sequence from Chr15::965998-966337 |  |
| pSR11-S6 | pSR11 plasmid with homologous sequence from Chr4::1166520-1166835 |  |
| pSR11-S2 | pSR11 plasmid with homologous sequence from Chr15::612582-612868 |  |
| pSR11-S4 | pSR11 plasmid with homologous sequence from Chr15::965998- 966332 |  |
| pSR11-S5 | pSR11 plasmid with homologous sequence from Chr4:: 916145-916404 |  |
|  |  |  |
| **Strain** | **Genotype** | **Note** |
| Background (yLB109) | MATa, *tor1-1*, *fpr1::NAT*, *bar1* |  |
| Pair 1 (yFZ102) | MATa, *tor1-1, fpr::NAT, bar1, HIS3::REV1pr-tetR-mCherry-REV1pr-LacI-GFP, ADE2::REV1pr-LacI-FKBP12-REV1pr-TetR-FRB, Chr15::433546_LacO, Chr15::793958_TetO* | Intra pair |
| Pair 2 (yFZ110) | MATa, *tor1-1, fpr::NAT, bar1, HIS3::REV1pr-tetR-mCherry-REV1pr-LacI-GFP, ADE2::REV1pr-LacI-FKBP12-REV1pr-TetR-FRB, Chr15::966175_LacO, Chr4::1166682_TetO* | Inter pair |
| Pair 3 (yFZ101) | *MATa, tor1-1, fpr::NAT, bar1, HIS3::REV1pr-tetR-mCherry-REV1pr-LacI-GFP, ADE2::REV1pr-LacI-FKBP12-REV1pr-TetR-FRB, Chr15::433546_LacO, Chr15::612715_TetO, MYO1-CFP-KanMx, NUP49-CFP-KanMx* | Intra pair |
| Pair 4 (yFZ103) | *MATa, tor1-1, fpr::NAT, bar1, HIS3::REV1pr-tetR-mCherry-REV1pr-LacI-GFP, ADE2::REV1pr-LacI-FKBP12-REV1pr-TetR-FRB, Chr15::433546_LacO, Chr15::966177_TetO, MYO1-CFP-KanMx, NUP49-CFP-KanMx* | Intra pair |
| Pair 5 (yFZ104) | *MATa, tor1-1, fpr::NAT, bar1, HIS3::REV1pr-tetR-mCherry-REV1pr-LacI-GFP, ADE2::REV1pr-LacI-FKBP12-REV1pr-TetR-FRB, Chr15::433546_LacO, Chr4::916282_TetO, MYO1-CFP-KanMx, NUP49-CFP-KanMx* | Inter Pair |
