# Supplementary material for: Hi-C calibration by chemically induced chromosomal interactions
Source: EMBO Rep. 2026 Apr 14;27(10):2549–58. doi: 10.1038/s44319-026-00772-x (PMC13219496; doi:10.1038/s44319-026-00772-x)
Supplement: Supplementary file 6 — Expanded View Figures [file 44319_2026_772_MOESM6_ESM.pdf]

Expanded View Figures

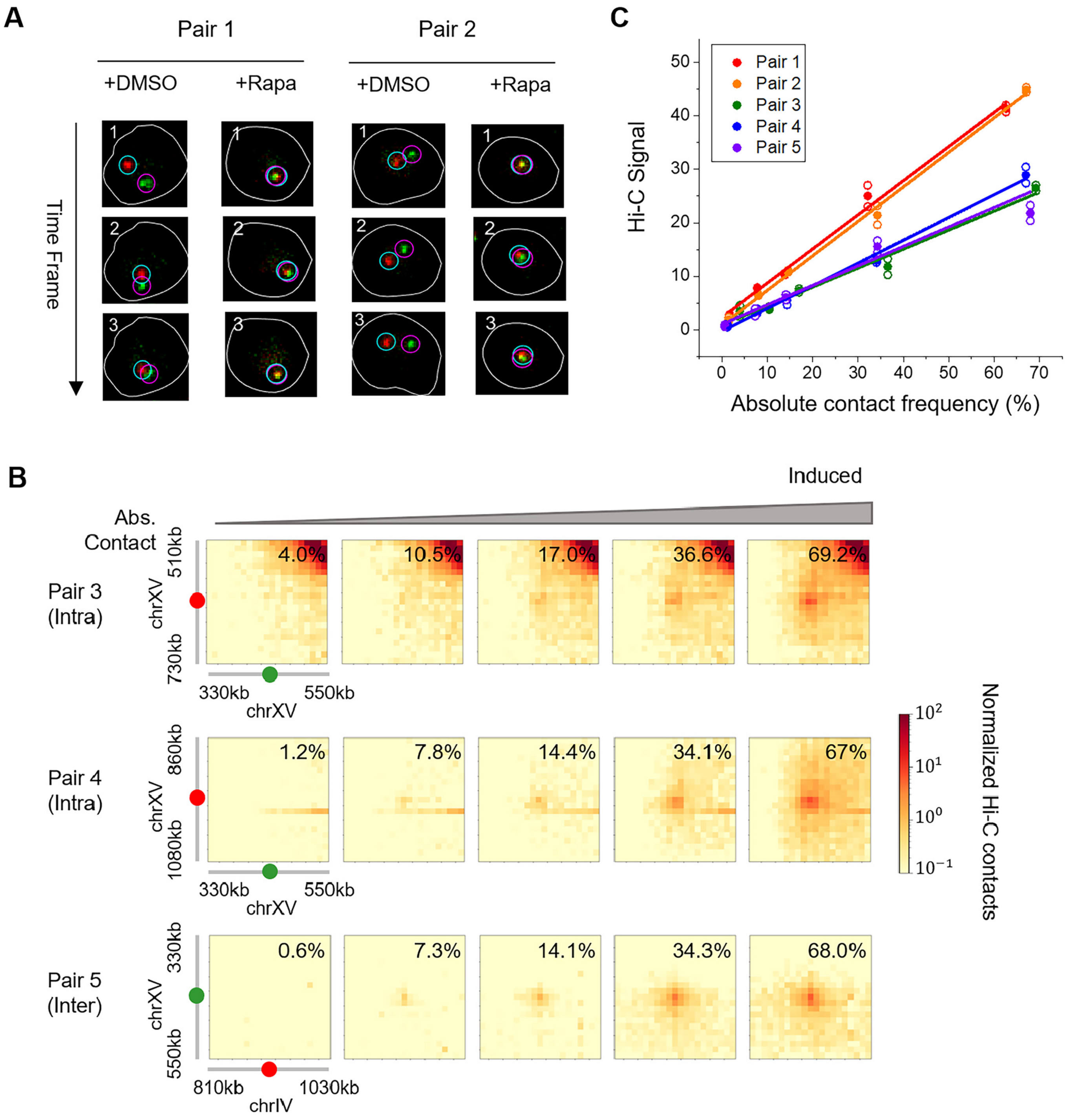

Figure EV1. Extra imaging and Hi-C data in CICI strains.

(**A**) Typical image data for CICI analysis. For each condition, annotated images of three consecutive frames are shown. Loci pairs are considered to be in contact if dots are continuously co-localized. (**B**) Hi-C data for pair 3–5 with titration of CICI cells. Hi-C data at the two end points (0 and 100% + rapamycin populations) are direct repeats from Fig. 2A. (**C**) Normalized but not corrected CICI signals as a function of absolute contact frequency.

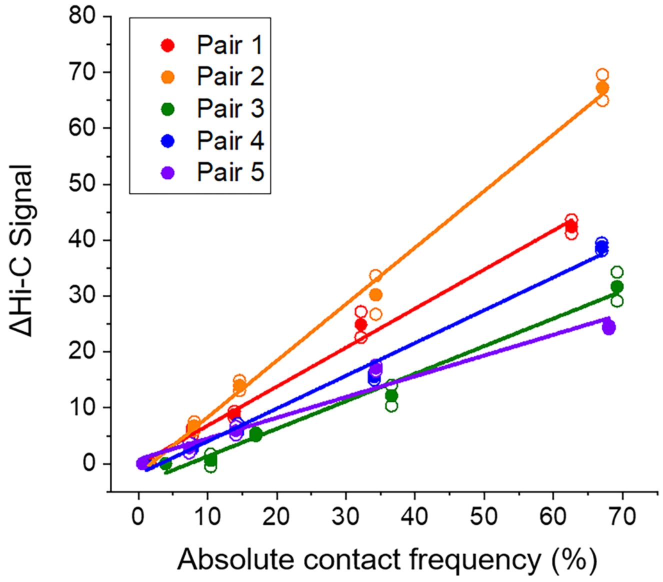

Figure EV2. Hi-C signal after background subtraction versus absolute contact frequency.

This plot is identical to Fig. 3C, except that the −rapamycin Hi-C signals (background) has been subtracted from the +rapamycin Hi-C signals.

 © The Author(s)
